# The human autophagy-initiating complexes ULK1C and PI3KC3-C1

**DOI:** 10.1016/j.jbc.2025.110391

**Published:** 2025-06-19

**Authors:** Minghao Chen, James H. Hurley

**Affiliations:** 1 Department of Molecular and Cell Biology, University of California, Berkeley, Berkeley, California, USA; 2 California Institute for Quantitative Biosciences, University of California, Berkeley, Berkeley, California, USA; 3 Aligning Science Across Parkinson's (ASAP) Collaborative Research Network, Chevy Chase, Maryland, USA; 4 Helen Wills Neuroscience Institute, University of California, Berkeley, Berkeley, California, USA

**Keywords:** autophagy, phosphoinositide kinase, protein kinase, ULK1, FIP200, ATG13, ATG101, VPS34, VPS15, BECN1, Beclin-1, ATG14, mitophagy, cryo-EM

## Abstract

The unc-51-like kinase complex (ULK1C) and the class III phosphatidylinositol 3-kinase complex I (PI3KC3-C1) are the key regulators of macroautophagy initiation. Understanding the assembly and coordination of these two complexes is essential for deciphering their cellular regulation and targeting them for therapeutic enhancement. This review highlights recent advances in our understanding of the structural organization and activation mechanisms of ULK1C and PI3KC3-C1 at the molecular level and discusses their roles within the protein interaction network governing autophagy initiation.

Macroautophagy, hereafter referred to as autophagy, is a fundamental process in eukaryotic cells that maintains homeostasis by degrading unnecessary or damaged cellular components. Dysfunction of autophagy is implicated in numerous human diseases, including cancer, inflammatory diseases, neurodegenerative disorders, and infections caused by viruses and bacteria (1, 2). Targeting the autophagy pathway has been recognized as a potential therapeutic strategy for a wide range of conditions (3, 4), including Parkinson’s disease (5).

Autophagy is classified into two types based on substrate selectivity: bulk (non-selective) autophagy and selective autophagy. Bulk autophagy, commonly triggered by nutrient deprivation, is regulated by key signaling pathways including the mammalian target of rapamycin complex 1 (mTORC1) and AMP-activated protein kinase (AMPK) (6, 7). This type of autophagy involves the degradation of cytoplasmic components, which are recycled in the lysosome into the building blocks of macromolecular synthesis. In contrast, selective autophagy targets specific substrates, such as protein aggregates (referred to as aggrephagy), mitochondria (mitophagy), lysosomes (lysophagy), the endoplasmic reticulum (ER-phagy), the Golgi (golgiphagy), nuclear pore complexes (NPC-phagy), peroxisomes (pexophagy), and intracellular pathogens (xenophagy) (8). Both forms of autophagy initiate with the *de novo* formation of a phagophore, also known as the isolation membrane—a double-membrane structure that sequesters the cargo (9, 10, 11, 12). Selective autophagy often begins with the ubiquitylation of cargo, which is subsequently recognized by ubiquitin-binding cargo receptors such as p62/SQSTM1, TAX1BP1, NDP52, NBR1, and OPTN (13, 14). These cargo receptors perform two main functions, (i) recruiting the autophagy machinery to the phagophore assembly site and initiating phagophore formation (Fig. 1*A*), and (ii) linking the cargo to phagophore through interactions with LC3/GABARAP proteins by their LC3-interaction region (LIR) motifs (15, 16, 17) (Fig. 1*B*).

**Figure 1:**
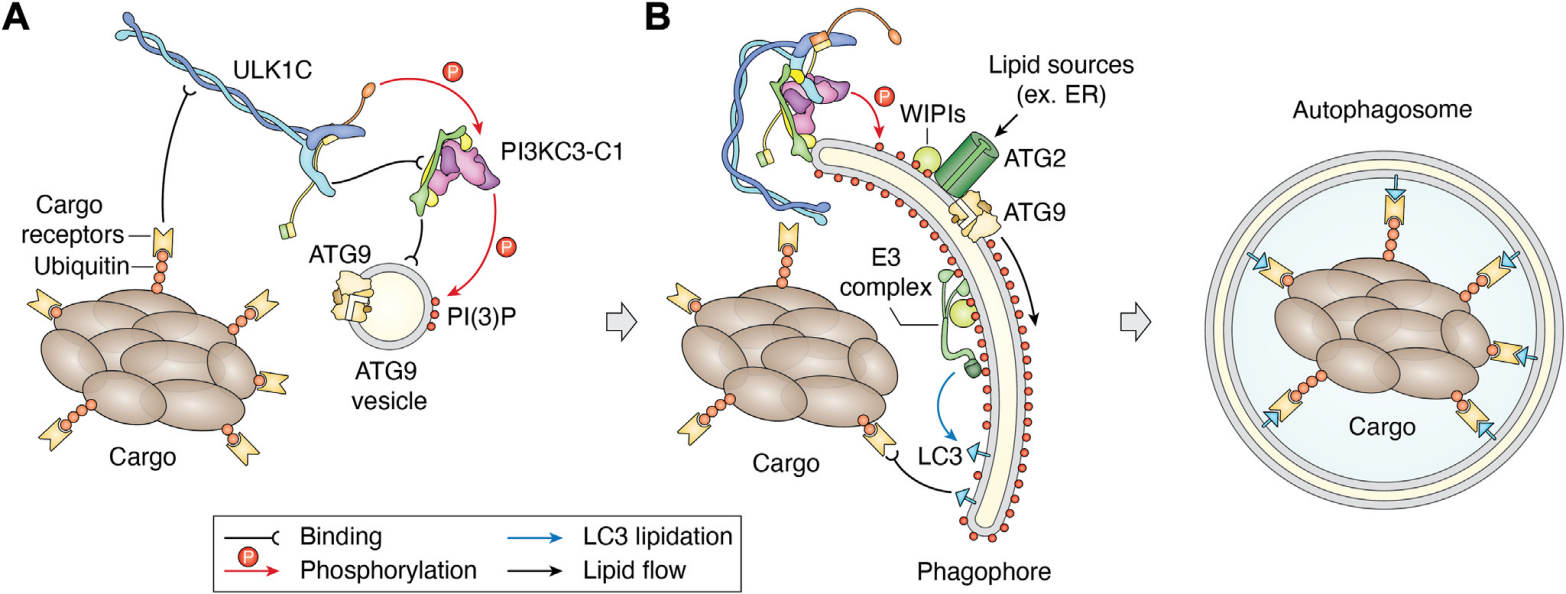
**Overview of autophagosome formation in selective autophagy**. *A*, initiation of phagophore formation. Selective autophagy cargos, such as protein aggregates, are first ubiquitinated and then recognized by specific cargo receptors. These cargo receptors subsequently recruit ULK1C, which in turn interacts with and activates PI3KC3-C1. PI3KC3-C1 produces PI(3)P on autophagy-associated membranes. *B*, phagophore expansion. PI(3)P recruits WIPI proteins, which facilitate the following recruitment of ATG2 and E3 complex. ATG2 transports lipids from donor organelles such as the ER, promoting phagophore expansion in collaboration with ATG9. Meanwhile, the E3 complex mediates the conjugation of LC3 proteins to the phagophore membrane, a process known as LC3 lipidation. Interactions between LC3 proteins and cargo receptors keep the phagophore growing around the cargo. After closure (also a critical step yet out of the scope of this review), the mature autophagosome is formed and subsequently fused with lysosomes for cargo degradation.

The unc-51-like kinase complex (ULK1C) is usually, but not always (18), the most upstream-acting of the core complexes, and responds to a variety of signals from both bulk and selective autophagy pathways. ULK1C transmits these signals by recruiting and activating downstream autophagy machinery (10, 19, 20) including the class III phosphatidylinositol 3-kinase complex I (PI3KC3-C1) (Fig. 1*A*). The primary function of PI3KC3-C1 is to produce phosphatidylinositol-3-phosphate (PI(3)P), which is a critical messenger in the autophagy signaling cascade that recruits effector WD repeat proteins interacting with phosphoinositides (WIPIs). WIPI proteins, in turn recruit two other autophagy core complexes: the ATG12-ATG5-ATG16L1 (E3) complex and ATG2A. The ATG12-ATG5-ATG16L1 has a ubiquitin E3-like activity in promoting ATG8 family protein conjugation to the membrane lipid phosphatidyl-ethanolamine (PE) and is often referred to as the “E3” in this context (Fig. 1*B*). Formation of the E3 complex is dependent on an ATG12-ATG5 conjugation reaction catalyzed by the ATG7 (E1-like) and ATG10 (E2-like) proteins. Meanwhile, ATG2A establishes contact sites between phagophores and the membrane sources, such as the ER, facilitating lipid transfer to the expanding phagophore (21, 22, 23) (Fig. 1*B*). The final autophagy core protein, ATG9A, acts as a scramblase within the phagophore membrane, balancing lipid distribution across the outer and inner leaflets downstream of ATG2A (24, 25, 26, 27, 28). Vesicles containing ATG9A, known as ATG9 vesicles, are proposed to serve as the initial seeds for phagophore formation (10, 29) (Fig. 1*A*).

In this review, we highlight recent advances in understanding the first two core complexes, ULK1C and PI3KC3-C1. The latest molecular studies on the structure, assembly, coordination, and activation of these complexes shed light on the mechanism underlying phagophore formation. These findings offer a detailed view of their regulation in atomistic detail and suggest concepts for their therapeutic targeting in the future.

## Overview of the ULK1 complex

**Figure 2:**
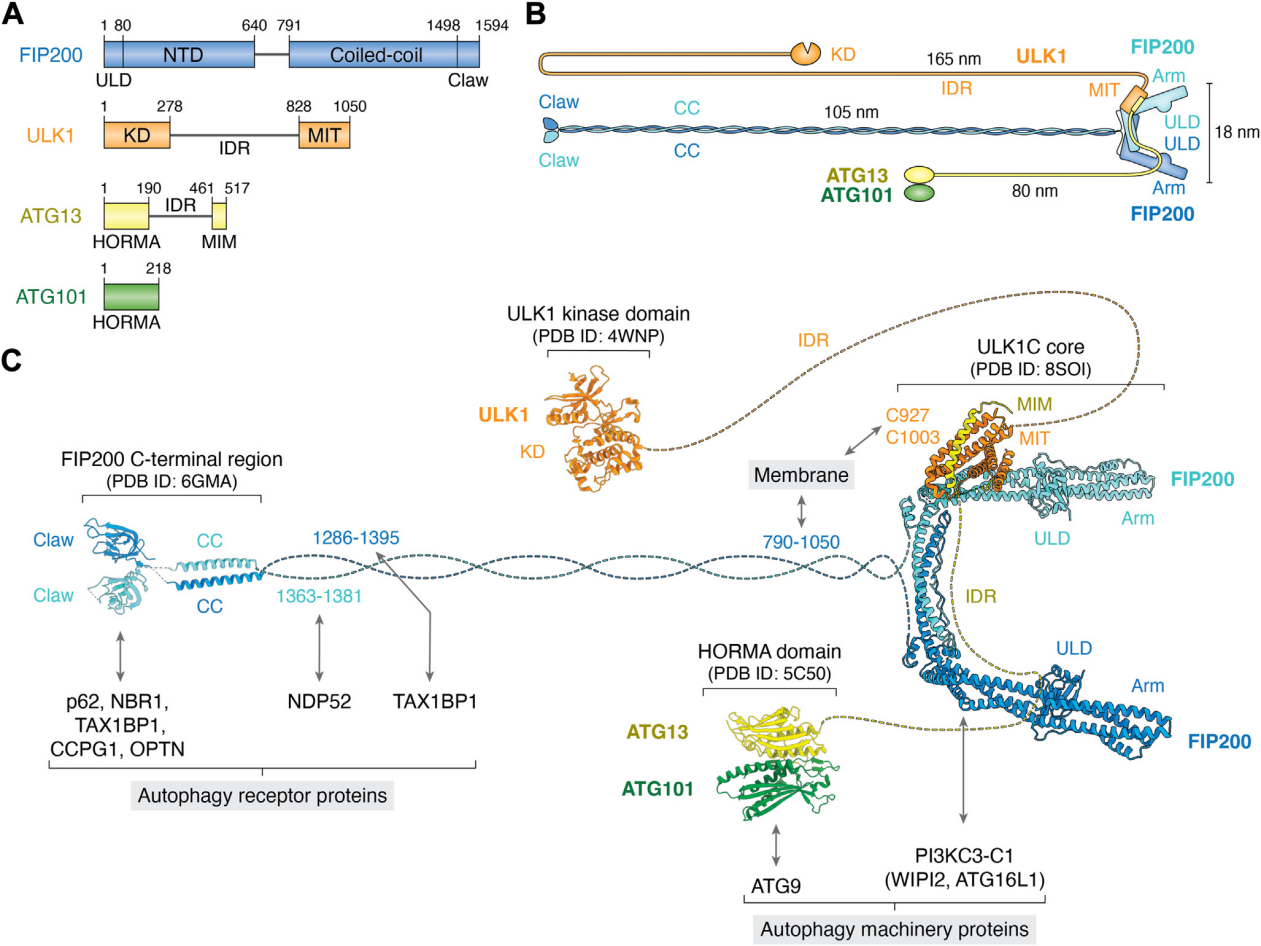
**Overall structure of ULK1C**. *A*, schematic representations for the subunits of ULK1C, with domains and their corresponding residue ranges indicated. *B*, schematic diagram of the overall structure of ULK1C, with each region shown to scale. The estimated contour lengths of the coiled-coil and IDRs are indicated. *C*, overview of the ULK1C generated from experimentally determined structures: core (PDB ID: 8SOI), ULK1 kinase domain (PDB ID: 4WNP), ATG13:ATG101 HORMA domain (PDB ID: 5C50), FIP200 C-terminal region (PDB ID: 6GMA). Domains and their interacting proteins are labeled.

The ULK1C is composed of four subunits: the serine/threonine protein kinase ULK1, the scaffold protein focal adhesion kinase family interacting protein of 200 kDa (FIP200), and the regulatory proteins autophagy-related protein 13 (ATG13) and 101 (ATG101) (Fig. 2*A*) (30, 31, 32, 33, 34). ULK1 is the catalytic subunit of the complex and comprises an N-terminal kinase domain (KD, residues 1–278), a long serine-proline-rich region (279–827), and a C-terminal tandem microtubule-interacting and transport (MIT) domain (828–1050), also sometimes referred to as the early autophagy tethering/targeting (EAT) domain (35). The N-terminal domain of ULK1 adopts a typical kinase structure but features a non-conserved regulatory loop, possessing an autophosphorylation site at Thr180 which is crucial for the autophagy activity (36). At the other end of ULK1, the C-terminal tandem MIT domain is responsible for interacting with other two ULK1C subunits FIP200 and ATG13 (37, 38). FIP200 consists of an N-terminal scaffold domain (NTD) (1–640) which contains within it an ubiquitin-like domain (ULD) (1, 2, 3, 4, 5, 6, 7, 8, 9, 10, 11, 12, 13, 14, 15, 16, 17, 18, 19, 20, 21, 22, 23, 24, 25, 26, 27, 28, 29, 30, 31, 32, 33, 34, 35, 36, 37, 38, 39, 40, 41, 42, 43, 44, 45, 46, 47, 48, 49, 50, 51, 52, 53, 54, 55, 56, 57, 58, 59, 60, 61, 62, 63, 64, 65, 66, 67, 68, 69, 70, 71, 72, 73, 74, 75, 76, 77, 78, 79, 80), an intrinsically disordered region (IDR, residues 641–790), a long coiled-coil (CC) domain (791–1497), and a C-terminal Claw domain (1498–1594). FIP200 forms a long, flexible homodimer that plays a central role in complex formation and interacts with various autophagy-related proteins (39). The remaining two subunits, ATG13 and ATG101, are primarily Hop1/Rev7/Mad2 (HORMA) proteins (40, 41). The N-terminal region of ATG13 (1–190) and the entire ATG101 (1–218) form a heteromeric HORMA dimer. This dimer connects to the core of ULK1C *via* an intrinsically disordered region (IDR) (191–460) and the C-terminal MIM motif (461–517) motif of ATG13 (38).

ULK1C is organized around a three-subunit core, which contains only a fraction of its total molecular mass. Three peripheral regions project from this core, each connected by a flexible linker (Fig. 2, *B* and *C*). The core region represents the largest ordered structure of the complex, consisting of FIP200^NTD^, ULK1^MIT^, and ATG13^MIM^. The FIP200^NTD^ forms a C-shaped homodimer, serving as a scaffold for recruiting the ULK1^MIT^: ATG13^MIM^ heterodimer *via* its ‘shoulder’ region. Basally, the ULK1C core has a 2:1:1 FIP200:ULK1:ATG13 stoichiometry (37, 38), in which the ATG13^IDR^ interacts extensively with both protomers of FIP200^NTD^ through two independent binding sites (ATG13^392-408^ and ATG13^450-460^). Truncations or mutations of these ATG13 IDR regions can change the subunit stoichiometry of the complex to 2:2:2 (38). The potential physiological implications of the core complex stoichiometry conversion will be discussed below. The peripheral regions of ULK1C include the ULK^KD^, the FIP200^Claw^, and the ATG13/101 HORMA dimer, which are linked to the core region through long coiled-coil or IDRs (Fig. 2*B*). The overall flexibility of ULK1C allows it to efficiently connect cargos and interact with the rest of the autophagy machinery over extended distances.

## FIP200 as an interaction hub

A key function of ULK1C is the recognition of cargo receptors, which triggers the initiation of selective autophagy (Fig. 2*C*, left). This role is, in most cases, attributable to the extreme C-terminal region of FIP200. The cargo receptor p62 recruits FIP200 through direct interaction with its claw domain (42), while another aggrephagy receptor, TAX1BP1, binds to both the CC and the claw domains of FIP200 (43, 44). Other cargo receptors, such as NBR1, also directly bind to FIP200 and enhance p62-mediated autophagy, although their specific binding sites on FIP200 remain to be identified (45, 46). The mitophagy and xenophagy receptor NDP52 interacts with both the FIP200^CC^ (1363–1381) and the FIP200^claw^ (47, 48). Moreover, the ER-phagy receptor CCPG1 and the mitophagy receptor OPTN engage FIP200 through the claw domain (49, 50). Besides cargo receptors, FIP200 also interacts with TNIP, a mitophagy regulator, *via* its claw domain (51, 52). While the FIP200 C-terminal region contains most of the documented cargo receptor binding sites, an alternative linkage has been reported for the mitophagy receptors BNIP3, NIX, and FKBP8 and the ER-phagy receptor TEX264. These receptors bind directly to WIPI2, which in turn recruits ULK1C through its ATG13 subunit (53).

In addition to the cargo receptors, FIP200 also interacts with various downstream autophagy proteins (Fig. 2*C*, right bottom). Among these, PI3KC3-C1 is the most crucial target of ULK1C, and their interaction is primarily mediated through FIP200 (38). FIP200 directly binds to ATG16L1 (54, 55), which is regulated by the phosphorylation of FIP200 and is critical for mitophagy (56). Moreover, FIP200 facilitates the ER-phagophore contact formation by binding to WIPI2, suggesting a positive feedback mechanism in autophagy initiation (57). Beyond direct cargo receptor interactions, FIP200 contributes to autophagy in various other ways: has been reported to interact with the ALS/FTD gene product C9orf72 (58), although the site of the interaction has not been defined, and reported to interact with WIPI3/TSC1 to regulate mTORC1 (59, 60), and associates with MOSPD2/VAPA/VAPB for localization to the autophagosome-ER contact site (61, 62). These interactions underscore the versatile function of FIP200 as an interaction hub in autophagy signaling, coordinating multiple pathways and processes essential for autophagy regulation.

The membrane association of FIP200 is a crucial event in autophagy initiation. Ectopic recruitment of FIP200 to mitochondria or peroxisomes results in the degradation of these organelles, bypassing the need for their endogenous receptors (47). This observation suggests that an increase in the local concentration of FIP200 is sufficient to induce autophagy. It has also been demonstrated that calcium transients trigger the condensate formation of FIP200 on ER in a ATG13-dependent manner, driving their assembly into phagophore assembly site (63). Neither FIP200 nor other subunits of ULK1C are known to be Ca^2+^-binding proteins, nor do they contain known Ca^2+^-binding motifs. It seems more likely this effect is mediated by Ca^2+^-regulated protein kinases (64). FIP200 can directly bind to membranes in a manner promoted by binding to the cargo receptor NDP52. NDP52 binding promotes a more dynamic conformation of FIP200^CC^ (790–1050), enhancing its membrane binding (39) (Fig. 2*C*, right top).

## ATG13:ATG101 HORMA heterodimer

The ATG13:ATG101 HORMA dimer is a distinct and characteristic component of ULK1C. ATG13 is conserved across eukaryotes, whereas ATG101 is absent in budding yeasts (31, 33). ATG101 contains only a HORMA domain and features a Trp-Phe (WF) finger motif, which is essential for autophagy but whose precise function and partner(s) are unknown (40, 41). ATG101 binds to the N-terminal HORMA domain of ATG13, which is connected to the ULK1C core *via* the IDR (37). The interaction between ATG13 and ATG101 is essential for autophagy (65). The HORMA domain is known for its unusual ability to switch between two different folds *via* a conformational change in its “seatbelt” region (66). It was proposed that the ATG13 and ATG101 HORMA domains can regulate autophagy by switching folds (67); however, this occurs on a time scale of many hours, far longer than the ∼2 min needed for autophagy initiation. As yet, neither experimental nor predictive structural evidence for this type of switching in the ATG13 and ATG101 context has been reported; thus, the concept of HORMA fold switching in autophagy must be considered speculative. On the other hand, high-resolution crystallography showed that a groove formed at the interface between the ATG13 and ATG101 HORMA domains binds to the C-terminal region of ATG9A (68). ATG9A C-terminal tail binding to the HORMA dimer is important, but not strictly required, for bulk autophagy and NDP52-dependent mitophagy (68).

## Regulation and activation of ULK1 kinase

ULK1 is the pivotal kinase in the autophagy initiation pathway, of which one primary substrate is ULK1 itself (36, 69) and the other three subunits of ULK1C (70, 71). ULK1 has many non-autophagic functions, which have been well-reviewed elsewhere and so will not be described further here (72). ULK1 phosphorylates PI3KC3-C1 (70, 73, 74) and its regulator AMBRA1 (70, 75), along with other autophagy-related proteins including the lipid scramblase ATG9A (76), the cysteine protease and LC3 deconjugase ATG4 (77), and others (20, 72). Meanwhile, ULK1 is also a phosphorylation target of upstream kinases such as mTORC1 (6), AMPK (6, 78, 79), and TIP60 (80). Under nutrient-rich conditions, ULK1 is inhibited by mTORC1, which phosphorylates key regulatory sites on ULK1 and ATG13, maintaining them in an inactive state (6, 81). In starvation conditions, these sites undergo rapid dephosphorylation, leading to the activation of ULK1 (30, 32). Remarkably, active ULK1 can phosphorylate and inhibit mTORC (82, 83), indicating a complex regulatory network involving feedback loops within nutrient-sensing signaling (7). The great majority of ULK1C phosphorylation sites are located within IDRs, such that to date, the structural mechanisms of phosphoregulation have yet to be explained.

**Figure 3:**
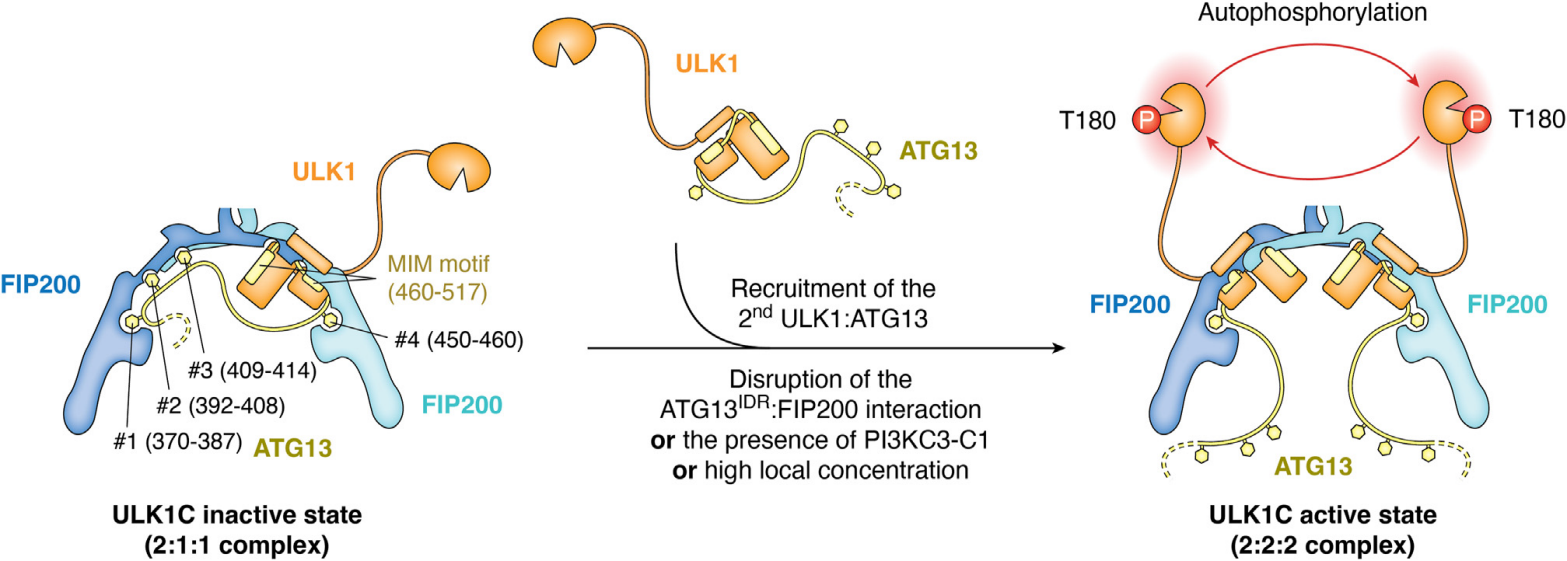
**Model of ULK1C activation.** Schematic diagram illustrating the activation process of ULK1C. In the inactive state, the ULK1C core forms a FIP200:ULK1:ATG13 complex with a 2:1:1 stoichiometry, where the ATG13^IDR^ extensively interacts with the FIP200^arm^ (sites #1–3), preventing the binding of a second ULK1:ATG13 subdimer (*left*). Alteration of stoichiometry can be induced by (i) increased local concentration or (ii) presence of PI3KC3-C1, which disrupts the ATG13:FIP200 interaction and facilitates the recruitment of the second ULK1:ATG13 subdimer to the core. This results in the formation of a 2:2:2 complex, enhancing the autophosphorylation of ULK1 (*right*).

Autophosphorylation is a critical step in ULK1 activation (36), though its molecular basis has been elusive. Recent findings propose a dimerization-induced activation mechanism of ULK1, based on the observed stoichiometry shift of FIP200:ULK1:ATG13 from 2:1:1 to 2:2:2. This mechanism parallels the well-known paradigm observed in receptor tyrosine kinases (RTKs) (84), and many other kinases, in which dimerization triggers autophosphorylation and activation (Fig. 3). This is consistent with the observation that artificially induced dimerization of Atg1, the yeast homologue of ULK1, promotes its activation (85). Multiple factors can drive this stoichiometry shift, including disruption of the interaction between ATG13^IDR^ and FIP200, increased concentration of ULK1C, and the presence of PI3KC3-C1. It is proposed that PI3KC3-C1 promotes the dimerization of ULK1C in part by increasing its local concentration at the phagophore assembly site, thereby facilitating its autophosphorylation and activation (38). Moreover, the N-termini of the BECN1 and ATG14 subunits of PI3KC3-C1 (see below) are positioned near the autoinhibitory ATG13^IDR^ binding sites of FIP200 (86), suggesting a potential regulatory role in this region.

In addition, ULK1 is directly anchored to the autophagosome formation site through palmitoylation of Cys927 and Cys1003 on the MIT domain. Depletion of the palmitoyl transferase ZDHHC13 impairs the recruitment of ULK1C and reduces the phosphorylation of ATG14, underscoring its role in the early steps of autophagy initiation (87).

## Overview of PI3KC3-C1

**Figure 4:**
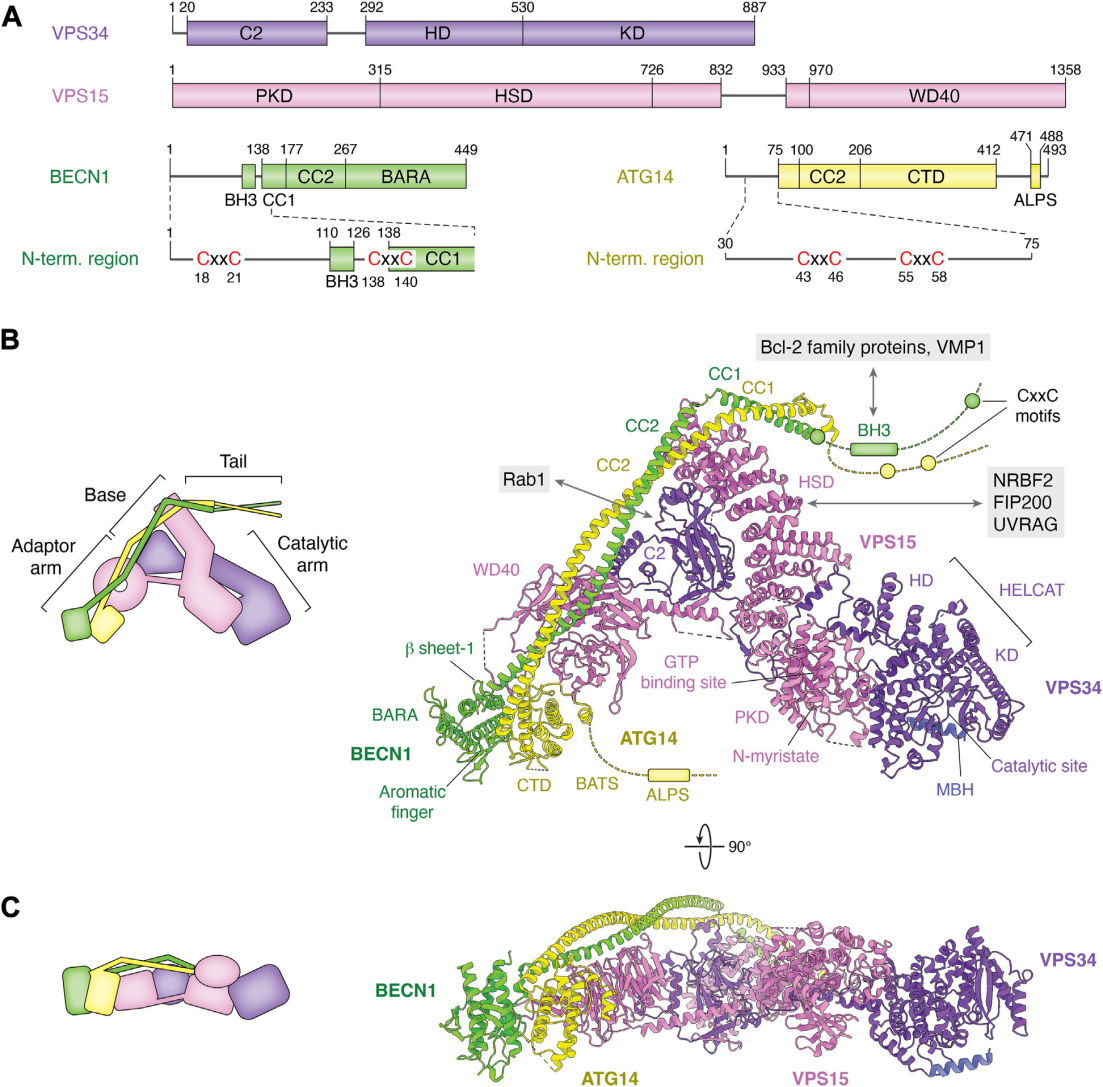
**Overall structure of PI3KC3-C1**. *A*, schematic representations for the subunits of PI3KC3-C1, with domains and their corresponding residue ranges indicated. *B*, *top* view of the active state of PI3KC3-C1 (PDB ID: 9MHH). Domains and their interacting proteins are labeled. A schematic diagram of the overall structure is shown in the *top-left* corner, with each region labeled: catalytic arm, adaptor arm, base, and tail. *C*, side view of the active PI3KC3-C1 structure, along with its corresponding schematic diagram.

PI3KC3-C1 is the second autophagy core complex, usually, but not always (18), functioning immediately downstream of ULK1C. This 360 kDa complex is composed of one copy each of four subunits: the lipid kinase vacuolar protein sorting 34 (VPS34), the regulatory subunit VPS15, and the membrane binding subunits BECN1 and ATG14, forming a 1:1:1:1 heterotetrametric assembly (88) (Fig. 4*A*). The kinase subunit VPS34 was first identified in budding yeast (89, 90, 91) and remains the sole member of the class III PI3K family across eukaryotes (92). The primary function of VPS34 is to produce PI(3)P, an essential lipid mediator in phagophore formation (93). The pseudokinase VPS15 was identified shortly after VPS34 as a constitutive binding partner essential for activity (94, 95, 96). Subsequent studies revealed that the N-terminal pseudokinase domain of VPS15 directly regulates the VPS34 kinase activity by sequestering its catalytic site (86, 97). BECN1 promotes autophagy (98) as a component of the PI3KC3-C1/C2 complexes (99) by providing a membrane binding site and interacting with various regulators, including Rubicon and AMBRA1 (100, 101). BECN1 is also a known binding target of the anti-apoptotic protein Bcl-2 mediated through its BH3 domain (102). ATG14 is the autophagy-specific subunit of PI3KC3-C1 (99, 103), and its phosphorylation at S29 by ULK1 is critical for autophagy initiation (74). ATG14 can be replaced by UV irradiation resistance-associated gene (UVRAG) while keeping the other three subunits (VPS34, VPS15, and BECN1) the same, to form the PI3KC3-C2 complex, which is central to endolysosomal sorting and also functions in later stages of autophagy (99, 104). The overall structure of the PI3KC3-C1 adopts a Y-shape conformation, which can be divided into a catalytic arm, an adaptor arm, a base, and a tail region (Fig. 4*B*). The catalytic domain constitutes only 11% of the total molecular mass of the complex, with the remaining 89% comprising non-catalytic components responsible for regulatory functions, including autoinhibition, activation, membrane targeting, and recruitment of other regulators. In the following sections, we will explore these regions in detail and discuss their respective functions.

## The catalytic arm

The catalytic arm is composed of the VPS34 kinase domain (VPS34^KD^), the VPS34 helical domain (VPS34^HD^), and the VPS15 pseudokinase domain (VPS15^PKD^). VPS34^KD^ is the “business end” of the complex in that it is directly responsible for the ATP-dependent phosphorylation of the 3′ position of phosphatidylinositol (PI). Structural studies of catalytic arm rearrangements have elucidated how VPS34^KD^ activity is regulated. The VPS34^KD^ and VPS34^HD^ together form the HELCAT (HELical and CATalytic) region initially described in crystallography studies (105, 106). VPS34^KD^ dynamics, or at least the ability of VPS34^KD^ to reposition itself, are essential for function in the context of PI3KC3-C1 (97, 107, 108, 109). In the inactive state, the catalytic site of VPS34^KD^ is sequestered by the adjacent VPS15^PKD^, rendering it inaccessible to membrane-localized PI. Conversely, in the active state, the VPS34^KD^ undergoes a 140-degree rotation, fully exposing the catalytic site to engage with PI in the membrane (86).

**Figure 5:**
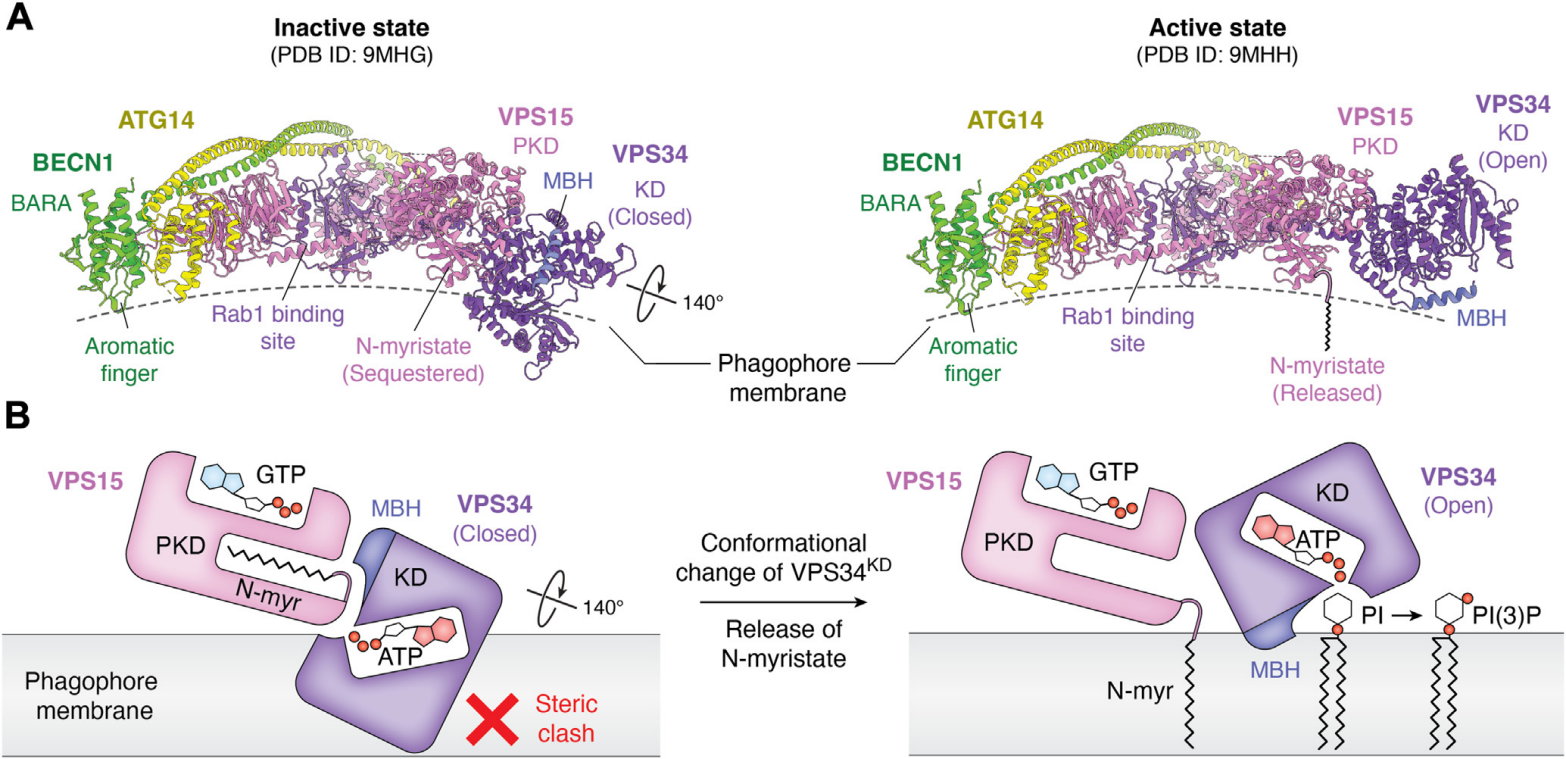
**Model of PI3KC3-C1 activation**. *A*, side view comparison of PI3KC3-C1 in its inactive (PDB ID: 9MHG) and active states (PDB ID: 9MHH). The membrane binding motifs and the presumed position of the membrane are indicated. *B*, schematic diagram illustrating the activation process of PI3KC3-C1. In the inactive state, the VPS34^KD^ clashes with the membrane. Upon activation, the N-myristate of VPS15^PKD^ is released from the pocket and inserted into the membrane, leading to a 140-degree rotation of VPS34^KD^. This conformational change allows VPS34^KD^ to fully associate with the membrane, facilitating catalytic activity. The relative positions of N-myristate (N-myr), membrane binding helix (MBH), ATP, and GTP/GDP are indicated.

This conformational shift is directly regulated by VPS15^PKD^, which acts as a scaffold extending from the base region of PI3KC3-C1 (Fig. 5). A single face of VPS15^PKD^ can bind to two distinct surfaces of VPS34^KD^. The nature of the interaction with VPS34^KD^ is regulated by the conformation of the *N*-myristoyl modification of VPS15. It has long been known that the N-terminus of VPS15 is myristoylated (94, 95, 110), but only recently appreciated that the VPS15 *N*-myristate plays an important role in regulating VPS34 (86). In the inactive state, the myristate is sequestered within the N-lobe of the VPS15^PKD^, stabilizing a series of interactions between VPS15^PKD^ and one face of VPS34^KD^ so as to hold VPS34^KD^ in the closed state. Upon activation, the myristate is liberated from its pocket, disrupting the original interactions and altering the binding preference to the open state. This mechanism is in some ways reminiscent of the myristoyl switch observed in Recoverin and ARF GTPases (111, 112, 113, 114), where the myristoyl moiety is sequestered in a hydrophobic pocket and released upon specific stimuli (*e*.*g*. Ca^2+^ binding or GTP exchange), enhancing the protein-membrane association by inserting the myristoyl chain into the lipid bilayer.

The myristate pocket of VPS15^PKD^ is adjacent to the “active site” nucleotide binding pocket in VPS15^PKD^, which is unique in the kinome in its specificity for GTP molecule over ATP (113, 114). VPS15^PKD^ has thus far not been demonstrated to be competent for GTP hydrolysis, suggesting that the GTP has a structural role, but is not necessarily turned over as part of its regulatory mechanism. To summarize, these observations suggest a complex regulatory interplay involving GTP, sequestration and release of the *N*-myristate, and a closed-open conformational change in regulating the ability of VPS34^KD^ to access its membrane substrate.

## The adaptor arm

The adaptor arm of PI3KC3-C1 comprises the VPS15 WD40 repeat domain (VPS15^WD40^), BECN1 beta-alpha repeat autophagy domain (BECN1^BARA^), ATG14 C-terminal domain (ATG14^CTD^), and ATG14 Bakor/ATG14 L autophagosome targeting sequence domains (ATG14^BATS^). The VPS15^WD40^ serves as a scaffold, while the BECN1^BARA^ and ATG14^CTD-BATS^ are responsible for membrane targeting. A hyperexposed aromatic finger of BECN1, consisting of Phe359, Phe360, and Trp361, acts as the primary membrane-binding site and is located at the tip of the adaptor arm (115, 116). In addition, Chang *et al*. identified a beta-sheet of BECN1 containing Phe270 and Phe274 that can undock from the BARA domain and insert into membranes. This secondary membrane binding site is a regulatory target in PI3KC3-C2 for at least three proteins: Rubicon, HIV-1 Nef, and Tat-BECN1 peptide (T-BP) (100).

The ATG14^BATS^ domain can also bind to membranes *via* a C-terminal amphipathic helix referred to as the amphipathic lipid packing sensor (ALPS), which does not recognize the curved geometry of membranes *per se*, but rather defects in lipid packing that arise from either membrane bending or other causes (117, 118, 119). ATG14^BATS^ is a transplantable motif that increases the affinity of PI3KC3 complexes for membranes (108) and enzyme activity (119).

## The base region

The base region of PI3KC3-C1 consists of the VPS34 C2 domain (VPS34^C2^), the VPS34 helical hairpin (VPS34^HH^) motif, and the VPS15 helical solenoid domain (VPS15^HSD^). This region provides a scaffold for various accessory proteins that regulate complex assembly and function. One of the key accessory proteins is the GTPase Rab1, which plays a crucial role in recruiting PI3KC3-C1 to the early phagophores (120). PI3KC3-C1 binds to Rab1 *via* a cleft formed by the VPS34^C2^ and VPS34^HH^, accommodating the GTP sensing switch-II region of Rab1 stabilized in the GTP-binding state (86). The VPS34^C2-HH^ pocket also mediates binding to Rab5 in PI3KC3-C2, while neither the PI3KC3-C1-specific component BECN1 nor the C2-specific component UVRAG directly interacts with Rab proteins (86, 120). While it is clear that Rab1 and Rab5 recognition contributes to the targeting and activation of PI3KC3-C1 and -C2, respectively, a complete structural explanation for the differential regulation of the two complexes remains to be obtained.

The VPS15^HSD^ provides the primary binding site for nuclear receptor binding factor 2 (NRBF2), which is often considered as the fifth component of PI3KC3-C1 (121) due to its strong binding affinity (K_d_ = 40 nM) (122) and its role in activity regulation (122, 123, 124). NRBF2 is a homodimeric protein and interacts with PI3KC3-C1 through its N-terminal domain (1, 2, 3, 4, 5, 6, 7, 8, 9, 10, 11, 12, 13, 14, 15, 16, 17, 18, 19, 20, 21, 22, 23, 24, 25, 26, 27, 28, 29, 30, 31, 32, 33, 34, 35, 36, 37, 38, 39, 40, 41, 42, 43, 44, 45, 46, 47, 48, 49, 50, 51, 52, 53, 54, 55, 56, 57, 58, 59, 60, 61, 62, 63, 64, 65, 66, 67, 68, 69, 70, 71, 72, 73, 74, 75, 76, 77, 78, 79, 80, 81, 82, 83, 84, 85, 86, 87, 88). Earlier studies demonstrated that NRBF2 induces the dimerization of PI3KC3-C1, forming a NRBF2:PI3KC3-C1 2:2 stoichiometry complex, where one NRBF2 dimer recruits two PI3KC3-C1 complexes (122, 125). However, subsequent research reported that an excess amount of NRBF2 is necessary to fully activate PI3KC3-C1, implying the presence of a secondary regulation site within the complex (109). NRBF2 is specific for PI3KC3-C1, consistent with the fact that its binding site on VPS15^HSD^ is blocked by the C2 domain of UVRAG in PI3KC3-C2 (121, 123, 126). Despite its tight binding, the availability of a moderate-resolution cryo-EM structure, and its evident functional importance, there is more yet to be learned concerning the role of NRBF2 in PI3KC3 regulation.

## The tail region

Although the overall structure of PI3KC3-C1 is often described as “V-shaped”, the complex indeed extends into a “Y-shape” due to the presence of a tail region composed of the N-termini of BECN1(1–136) and ATG14 (1, 2, 3, 4, 5, 6, 7, 8, 9, 10, 11, 12, 13, 14, 15, 16, 17, 18, 19, 20, 21, 22, 23, 24, 25, 26, 27, 28, 29, 30, 31, 32, 33, 34, 35, 36, 37, 38, 39, 40, 41, 42, 43, 44, 45, 46, 47, 48, 49, 50, 51, 52, 53, 54, 55, 56, 57, 58, 59, 60, 61, 62, 63, 64, 65, 66, 67, 68, 69, 70, 71, 72). The tail is not visualized in most cryo-EM structures due to intrinsic disorder (127), but it harbors multiple motifs that play essential roles in the regulation of PI3KC3-C1, and it has been visualized at moderate resolution in the context of the supercomplex with ULK1C (38).

The BH3 motif (BECN1^BH3^, residues 110–126), is a key regulatory element within the tail region (128). Under nutrient-rich conditions, the BECN1^BH3^ is sequestered by Bcl-2 family proteins, thereby preventing the formation of functional PI3KC3-C1 and suppressing autophagy (129). In contrast, under starvation conditions, the BECN1^BH3^ is released either by phosphorylation of Bcl-2 family proteins or by competitive displacement by other BH3 proteins, leading to autophagy activation (130, 131, 132). The ULK1C:PI3KC3-C1 supercomplex structure revealed that the BECN1^BH3^ contributes to supercomplex formation through interaction with FIP200, suggesting the accessibility of BECN1^BH3^ could influence its ability to interact with ULK1C in autophagy initiation (38). BECN1^BH3^ is protected from hydrogen-deuterium exchange upon binding with proautophagic protein NRBF2 (122). These seemingly contradictory observations call for further investigation.

Both BECN1 and ATG14 contain two CxxC motifs at their N-termini, which are highly conserved across all eukaryotes (133). The CxxC motifs in BECN1 (Cys18-Cys21 and Cys137-Cys140) are responsible for coordinating Zn^2+^ ions and are critical for starvation-induced autophagy (134). In ATG14, the two CxxC motifs (Cys43-Cys46 and Cys55-Cys58) are essential for its localization on the ER (135). Our recent cryo-EM structure revealed one CxxC motif from BECN1 (Cys137-Cys140) and one from ATG14 (Cys43-Cys46) form a heterogeneous Zn-finger positioned behind the BH3 domain, supporting its interaction with FIP200 (38). The motif swapping and their resulting Zn-finger formation raise the possibility of a regulatory role in the functions of PI3KC3-C1.

## Activation of PI3KC3-C1

Understanding the activation of PI3KC3-C1 is one of the central questions for elucidating autophagy initiation. Extensive research has focused on identifying the post-translational modifications of PI3KC3-C1, including phosphorylation (88), myristoylation (94, 95), acetylation (136, 137), palmitoylation (138), and SUMOylation (139). Among these, phosphorylation has been particularly well-studied, significantly advancing our knowledge of the autophagy signaling pathways involving the kinases mTORC, ULK1, AMPK, Akt, and DAPK, among others (140, 141, 142, 143, 144). Most of these modifications have been characterized at the cellular level, but the precise molecular mechanism remains elusive in most cases because most of the phosphosites are on intrinsically disordered loops and distant from the catalytic center and known accessory protein binding sites.

Enhancing the membrane association properties of PI3KC3-C1 is a direct mechanism for its activation. The BECN1^BARA^ and ATG14^BATS^ domains play primary roles in membrane interaction and curvature sensing (115, 117). Moreover, the small GTPase Rab1 has been demonstrated to activate PI3KC3-C1 by recruiting it to target membranes (120), but also by biasing its overall conformation towards the active state (86). The interaction between the VPS34^KD^ and VPS15^PKD^ is at the crux of PI3KC3 activation (97, 106, 109, 120). The active, open conformation of PI3KC3-C1 is characterized by the exposure of both the catalytic site of VPS34^KD^ and the myristoylated N-terminus of VPS15. Given the observation that (i) PI3KC3-C1 adopts a flat overall structure in the open state (Fig. 4*C*) and (ii) all the above-mentioned sites are on the same side of the PI3KC3-C1 complex, a model for membrane docking was developed that presents an extensive surface to the membrane (86). This model was validated by all-atom molecular dynamics (MD) simulations (86), allowing all the above-mentioned membrane binding elements to simultaneously associate with the target membrane (Fig. 5).

The BH3 domain of BECN1 is a long-studied region for regulating the activity of PI3KC3-C1, primarily through its interaction with Bcl-2 family proteins including Bcl-2, Bcl-X_L_, Bcl-w, and Mcl-1 (129, 145). The interaction between BECN1 and Bcl-2 is regulated by various proteins. Activating molecule in BECN1-regulated autophagy protein 1 (AMBRA1) is a pro-autophagy protein that competes with Bcl-2 for binding to BECN1 (146). Upon starvation, AMBRA1 is phosphorylated by the ULK1C, which enhances the formation of the AMBRA1-BECN1 complex and facilitates its relocation to the phagophore assembly site to participate in autophagy initiation (75, 147). Additionally, the ER-localized transmembrane protein, vacuole membrane protein 1 (VMP1), is reported to interact with the BH3 domain of BECN1, releasing it from Bcl-2 and promoting the autophagy initiation (148, 149). The various reports of cellular relocalization of PI3KC3-C1 driven by BECN1^BH3^ will ideally be further corroborated by biochemical and structural analyses in the coming years.

## Crosstalk between ULK1C and PI3KC3-C1

**Figure 6:**
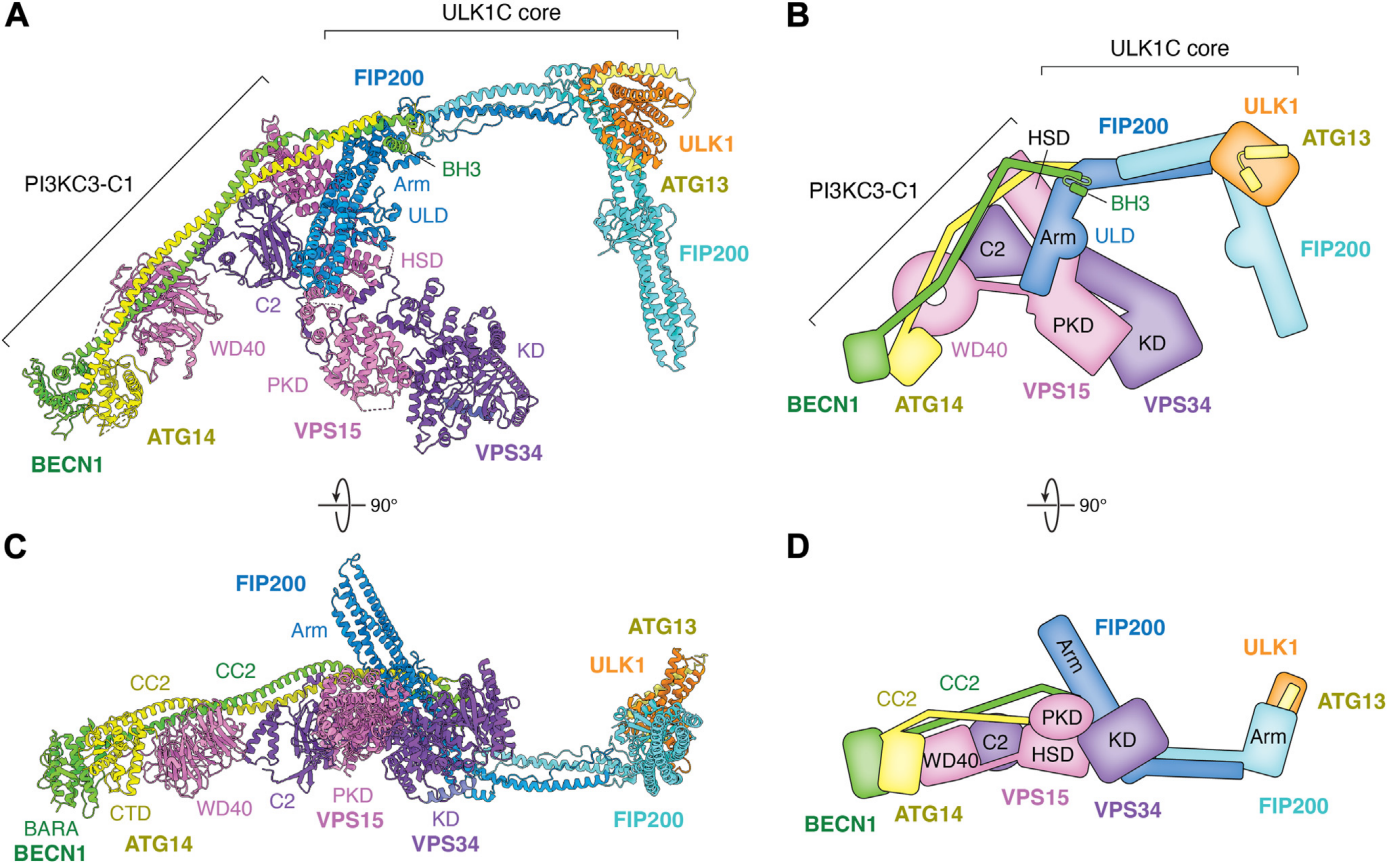
**Interface between ULK1C and PI3KC3-C1**. *A*, *top* view of the ULK1C:PI3KC3-C1 supercomplex model, generated by superposing the moderate-resolution supercomplex structure (PDB ID: 9C82) with the high-resolution structures of ULK1C core (PDB ID: 8SOI) and PI3KC3-C1 active state (PDB ID: 9MHH). Each subunit and its corresponding domains are labeled. *B*, Schematic diagram of *panelA*. *C* and *D*, side view of the supercomplex model and its schematic diagram.

As the two most upstream core complexes in the autophagy signaling pathway, the interplay between ULK1C and PI3KC3-C1 is thought to be crucial for phagophore formation. ULK1 directly phosphorylates all the four subunits of PI3KC3-C1, enhancing its activity and increasing the production of PI(3)P (70, 73, 74, 150, 151, 152). ULK1 kinase phosphorylates key regulators of PI3KC3-C1, including mTORC1, AMPK, and AMBRA1, which eventually impinge upon PI3KC3-C1 activity (75, 140, 142). In addition to phosphorylation, ULK1C physically interacts with PI3KC3-C1, which could potentially facilitate the recruitment of PI3KC3-C1 to the phagophore assembly site. The two complexes interact *via* the FIP200 arm region of ULK1C and the VPS15^HSD^-tail regions of PI3KC3-C1 (38) (Fig. 6). The interaction between ULK1C and PI3KC3-C1 extends beyond a simple one-directional regulation. Our recent study observed a shift in the stoichiometry of ULK1C from 2:1:1 to 2:2:2 in the presence of PI3KC3-C1 (38). By analogy to the dimerization-linked activation of many other kinases, the dimerization of ULK1 in this context could, in principle, trigger autophosphorylation. ULK1 autophosphorylation at Thr180 is known to be required for ULK1 kinase activity (36). ULK1 initiates autophagy within puncta containing more than 30 molecules that are co-localized with PI3KC3-C1 (153). It is attractive to speculate that membrane binding by PI3KC3-C1 and ULK1C mutually increases their local concentration and activity at the phagophore assembly site.

## Summary and outlook

ULK1C and PI3KC3-C1 are pivotal in the autophagy initiation. Recent advances in our understanding of these two core complexes have shed light on their intricate regulation and activation mechanisms. ULK1C is not only the most upstream kinase in the phagophore formation signaling pathway under most circumstances, but also a versatile scaffold that bridges cargo receptors to the rest of the core autophagy machinery. Autophosphorylation is critical for ULK1 kinase activation, potentially enhanced through dimerization of ULK1 on the scaffold subunit FIP200. Conversely, PI3KC3-C1 functions as a crucial lipid kinase downstream of ULK1. Its activity is modulated by conformational change of the VPS34^KD^ domain, which enhances membrane association and facilitates the production of PI(3)P on phagophore.

The initiation of phagophore formation is typically described as a hierarchical process, beginning with ULK1C activating PI3KC3-C1, which then recruits the PI(3)P effector WIPI proteins, followed by the assembly of ATG9A, ATG2A and the ATG12-ATG5-ATG16L1 complex. It is becoming clear that this process is far from a simple one-directional cascade. Recent reports have uncovered multiple feedback loops, such as the FIP200-ATG16 interaction (56), the ULK1-GABARAP interaction (154), the PI3K-GABARAP interaction (15), PI3KC3-C1-TBK1 (18), and the PI3KC3-C1-WIPI2 interaction (155), Our recent findings on the interplay between ULK1C and PI3KC3-C1 have introduced another feedback mechanism that alters the stoichiometry of ULK1C, providing new insights into this intricate and sophisticated network.

High-resolution structures of ULK1C core, PI3KC3-C1, and their supercomplex have revealed amino acid-level details of these complexes, highlighting several potential regulatory sites. Notable sites include the FIP200^NTD^: ATG13^IDR^ and the FIP200^NTD^: PI3KC3-C1 interaction regions, which are critical for ULK1 activation, as well as the *N*-myristate pocket and the GTP binding site on VPS15^PKD^, which are pivotal for PI3KC3-C1 activation. These newly identified regulatory sites present attractive targets for pharmaceutical development, potentially enabling the modulation of autophagy initiation as a therapeutic strategy.

## Conflict of interest

The authors declare the following financial interests/personal relationships which may be considered as potential competing interests: J. H. H. is a cofounder of Casma Therapeutics and receives research funding from Hoffmann-La Roche. M.C. declares that he has no competing interests.

## References

[1] Mizushima N., Levine B. (2020). Autophagy in human diseases. N. Engl. J. Med..

[2] Themistokleous C., Bagnoli E., Parulekar R., Muqit M.M.K. (2023). Role of autophagy pathway in Parkinson's disease and related genetic neurological disorders. J. Mol. Biol..

[3] Galluzzi L., Bravo-San Pedro J.M., Levine B., Green D.R., Kroemer G. (2017). Pharmacological modulation of autophagy: therapeutic potential and persisting obstacles. Nat. Rev. Drug. Discov..

[4] Onorati A.V., Dyczynski M., Ojha R., Amaravadi R.K. (2018). Targeting autophagy in cancer. Cancer.

[5] Antico O., Thompson P.W., Hertz N.T., Muqit M.M.K., Parton L.E. (2025). Targeting mitophagy in neurodegenerative diseases. Nat. Rev. Drug. Discov..

[6] Kim J., Kundu M., Viollet B., Guan K.L. (2011). AMPK and mTOR regulate autophagy through direct phosphorylation of Ulk1. Nat. Cell. Biol..

[7] Alers S., Loffler A.S., Wesselborg S., Stork B. (2012). Role of AMPK-mTOR-Ulk1/2 in the regulation of autophagy: cross talk, shortcuts, and feedbacks. Mol. Cell. Biol..

[8] Lamark T., Johansen T. (2021). Mechanisms of selective autophagy. Annu. Rev. Cell. Dev. Biol..

[9] Nakatogawa H. (2020). Mechanisms governing autophagosome biogenesis. Nat. Rev. Mol. Cell. Biol..

[10] Melia T.J., Lystad A.H., Simonsen A. (2020). Autophagosome biogenesis: from membrane growth to closure. J. Cell. Biol..

[11] Chang C., Jensen L.E., Hurley J.H. (2021). Autophagosome biogenesis comes out of the black box. Nat. Cell. Biol..

[12] Hurley J.H., Young L.N. (2017). Mechanisms of autophagy initiation. Annu. Rev. Biochem..

[13] Adriaenssens E., Ferrari L., Martens S. (2022). Orchestration of selective autophagy by cargo receptors. Curr. Biol..

[14] Vargas J.N.S., Hamasaki M., Kawabata T., Youle R.J., Yoshimori T. (2023). The mechanisms and roles of selective autophagy in mammals. Nat. Rev. Mol. Cell. Biol..

[15] Birgisdottir A.B., Mouilleron S., Bhujabal Z., Wirth M., Sjottem E., Evjen G. (2019). Members of the autophagy class III phosphatidylinositol 3-kinase complex I interact with GABARAP and GABARAPL1 via LIR motifs. Autophagy.

[16] Kirkin V., McEwan D.G., Novak I., Dikic I. (2009). A role for ubiquitin in selective autophagy. Mol. Cell..

[17] Kirkin V., Rogov V.V. (2019). A diversity of selective autophagy receptors determines the specificity of the autophagy pathway. Mol. Cell..

[18] Nguyen T.N., Sawa-Makarska J., Khuu G., Lam W.K., Adriaenssens E., Fracchiolla D. (2023). Unconventional initiation of PINK1/Parkin mitophagy by Optineurin. Mol. Cell..

[19] Wesselborg S., Stork B. (2015). Autophagy signal transduction by ATG proteins: from hierarchies to networks. Cell. Mol. Life. Sci..

[20] Zachari M., Ganley I.G. (2017). The mammalian ULK1 complex and autophagy initiation. Essays. Biochem..

[21] Maeda S., Otomo C., Otomo T. (2019). The autophagic membrane tether ATG2A transfers lipids between membranes. Elife.

[22] Osawa T., Kotani T., Kawaoka T., Hirata E., Suzuki K., Nakatogawa H. (2019). Atg2 mediates direct lipid transfer between membranes for autophagosome formation. Nat. Struct. Mol. Biol..

[23] Valverde D.P., Yu S., Boggavarapu V., Kumar N., Lees J.A., Walz T. (2019). ATG2 transports lipids to promote autophagosome biogenesis. J. Cell. Biol..

[24] Gomez-Sanchez R., Rose J., Guimaraes R., Mari M., Papinski D., Rieter E. (2018). Atg9 establishes Atg2-dependent contact sites between the endoplasmic reticulum and phagophores. J. Cell. Biol..

[25] Maeda S., Yamamoto H., Kinch L.N., Garza C.M., Takahashi S., Otomo C. (2020). Structure, lipid scrambling activity and role in autophagosome formation of ATG9A. Nat. Struct. Mol. Biol..

[26] Matoba K., Kotani T., Tsutsumi A., Tsuji T., Mori T., Noshiro D. (2020). Atg9 is a lipid scramblase that mediates autophagosomal membrane expansion. Nat. Struct. Mol. Biol..

[27] Guardia C.M., Tan X.F., Lian T., Rana M.S., Zhou W., Christenson E.T. (2020). Structure of human ATG9A, the only transmembrane protein of the core autophagy machinery. Cell. Rep..

[28] van Vliet A.R., Chiduza G.N., Maslen S.L., Pye V.E., Joshi D., De Tito S. (2022). ATG9A and ATG2A form a heteromeric complex essential for autophagosome formation. Mol. Cell..

[29] Cook A.S.I., Hurley J.H. (2023). Toward a standard model for autophagosome biogenesis. J. Cell. Biol..

[30] Ganley I.G., Lam du H., Wang J., Ding X., Chen S., Jiang X. (2009). ULK1.ATG13.FIP200 complex mediates mTOR signaling and is essential for autophagy. J. Biol. Chem..

[31] Hosokawa N., Sasaki T., Iemura S., Natsume T., Hara T., Mizushima N. (2009). Atg101, a novel mammalian autophagy protein interacting with Atg13. Autophagy.

[32] Jung C.H., Jun C.B., Ro S.H., Kim Y.M., Otto N.M., Cao J. (2009). ULK-Atg13-FIP200 complexes mediate mTOR signaling to the autophagy machinery. Mol. Biol. Cell..

[33] Mercer C.A., Kaliappan A., Dennis P.B. (2009). A novel, human Atg13 binding protein, Atg101, interacts with ULK1 and is essential for macroautophagy. Autophagy.

[34] Lin M.G., Hurley J.H. (2016). Structure and function of the ULK1 complex in autophagy. Curr. Opin. Cell. Biol..

[35] Ragusa M.J., Stanley R.E., Hurley J.H. (2012). Architecture of the Atg17 complex as a scaffold for autophagosome biogenesis. Cell.

[36] Lazarus M.B., Novotny C.J., Shokat K.M. (2015). Structure of the human autophagy initiating kinase ULK1 in complex with potent inhibitors. ACS. Chem. Biol..

[37] Shi X., Yokom A.L., Wang C., Young L.N., Youle R.J., Hurley J.H. (2020). ULK complex organization in autophagy by a C-shaped FIP200 N-terminal domain dimer. J. Cell. Biol..

[38] Chen M., Ren X., Nguyen T.N., Khuu G., Cook A.S.I., Lazarou M. (2025). Structure and activation of the human autophagy-initiating ULK1C:PI3KC3-C1 supercomplex. Nat. Struct. Mol. Biol..

[39] Shi X., Chang C., Yokom A.L., Jensen L.E., Hurley J.H. (2020). The autophagy adaptor NDP52 and the FIP200 coiled-coil allosterically activate ULK1 complex membrane recruitment. Elife.

[40] Qi S., Kim D.J., Stjepanovic G., Hurley J.H. (2015). Structure of the human atg13-atg101 HORMA heterodimer: an interaction hub within the ULK1 complex. Structure.

[41] Suzuki H., Kaizuka T., Mizushima N., Noda N.N. (2015). Structure of the Atg101-Atg13 complex reveals essential roles of Atg101 in autophagy initiation. Nat. Struct. Mol. Biol..

[42] Turco E., Witt M., Abert C., Bock-Bierbaum T., Su M.Y., Trapannone R. (2019). FIP200 claw domain binding to p62 promotes autophagosome formation at ubiquitin condensates. Mol. Cell..

[43] Zhang M., Wang Y., Gong X., Wang Y., Zhang Y., Tang Y. (2024). Mechanistic insights into the interactions of TAX1BP1 with RB1CC1 and mammalian ATG8 family proteins. Proc. Natl. Acad. Sci. U. S. A..

[44] Ohnstad A.E., Delgado J.M., North B.J., Nasa I., Kettenbach A.N., Schultz S.W. (2020). Receptor-mediated clustering of FIP200 bypasses the role of LC3 lipidation in autophagy. EMBO J..

[45] Ravenhill B.J., Boyle K.B., von Muhlinen N., Ellison C.J., Masson G.R., Otten E.G. (2019). The cargo receptor NDP52 initiates selective autophagy by recruiting the ULK complex to cytosol-invading bacteria. Mol. Cell..

[46] Turco E., Savova A., Gere F., Ferrari L., Romanov J., Schuschnig M. (2021). Reconstitution defines the roles of p62, NBR1 and TAX1BP1 in ubiquitin condensate formation and autophagy initiation. Nat. Commun..

[47] Vargas J.N.S., Wang C., Bunker E., Hao L., Maric D., Schiavo G. (2019). Spatiotemporal control of ULK1 activation by NDP52 and TBK1 during selective autophagy. Mol. Cell..

[48] Fu T., Zhang M., Zhou Z., Wu P., Peng C., Wang Y. (2021). Structural and biochemical advances on the recruitment of the autophagy-initiating ULK and TBK1 complexes by autophagy receptor NDP52. Sci. Adv..

[49] Smith M.D., Harley M.E., Kemp A.J., Wills J., Lee M., Arends M. (2018). CCPG1 is a non-canonical autophagy cargo receptor essential for ER-phagy and pancreatic ER proteostasis. Dev. Cell..

[50] Zhou Z., Liu J., Fu T., Wu P., Peng C., Gong X. (2021). Phosphorylation regulates the binding of autophagy receptors to FIP200 Claw domain for selective autophagy initiation. Nat. Commun..

[51] Le Guerroue F., Bunker E.N., Rosencrans W.M., Nguyen J.T., Basar M.A., Werner A. (2023). TNIP1 inhibits selective autophagy via bipartite interaction with LC3/GABARAP and TAX1BP1. Mol. Cell..

[52] Wu S., Li M., Wang L., Yang L., Cui J., Li F. (2024). Structural basis for TNIP1 binding to FIP200 during mitophagy. J. Biol. Chem..

[53] Adriaenssens E., Schaar S., Cook A.S.I., Stuke J.F.M., Sawa-Makarska J., Nguyen T.N. (2024). Reconstitution of BNIP3/NIX-mediated autophagy reveals two pathways and hierarchical flexibility of the initiation machinery. Nat. Cell. Biol..

[54] Gammoh N., Florey O., Overholtzer M., Jiang X. (2013). Interaction between FIP200 and ATG16L1 distinguishes ULK1 complex-dependent and -independent autophagy. Nat. Struct. Mol. Biol..

[55] Nishimura T., Kaizuka T., Cadwell K., Sahani M.H., Saitoh T., Akira S. (2013). FIP200 regulates targeting of Atg16L1 to the isolation membrane. EMBO Rep..

[56] Eickhorst C., Babic R., Rush-Kittle J., Lucya L., Imam F.L., Sanchez-Martin P. (2024). FIP200 phosphorylation regulates late steps in mitophagy. J. Mol. Biol..

[57] Zhao Y.G., Chen Y., Miao G., Zhao H., Qu W., Li D. (2017). The ER-localized transmembrane protein EPG-3/VMP1 regulates SERCA activity to control ER-isolation membrane contacts for autophagosome formation. Mol. Cell..

[58] Webster C.P., Smith E.F., Bauer C.S., Moller A., Hautbergue G.M., Ferraiuolo L. (2016). The C9orf72 protein interacts with Rab1a and the ULK1 complex to regulate initiation of autophagy. EMBO J..

[59] Gan B., Melkoumian Z.K., Wu X., Guan K.L., Guan J.L. (2005). Identification of FIP200 interaction with the TSC1-TSC2 complex and its role in regulation of cell size control. J. Cell. Biol..

[60] Bakula D., Muller A.J., Zuleger T., Takacs Z., Franz-Wachtel M., Thost A.K. (2017). WIPI3 and WIPI4 beta-propellers are scaffolds for LKB1-AMPK-TSC signalling circuits in the control of autophagy. Nat. Commun..

[61] Di Mattia T., Martinet A., Ikhlef S., McEwen A.G., Nomine Y., Wendling C. (2020). FFAT motif phosphorylation controls formation and lipid transfer function of inter-organelle contacts. EMBO J..

[62] Zhao Y.G., Zhang H. (2018). Formation and maturation of autophagosomes in higher eukaryotes: a social network. Curr. Opin. Cell. Biol..

[63] Zheng Q., Chen Y., Chen D., Zhao H., Feng Y., Meng Q. (2022). Calcium transients on the ER surface trigger liquid-liquid phase separation of FIP200 to specify autophagosome initiation sites. Cell.

[64] Zheng Q., Zhang H., Zhao H., Chen Y., Yang H., Li T. (2025). Ca(2+)/calmodulin-dependent protein kinase II beta decodes ER Ca(2+) transients to trigger autophagosome formation. Mol. Cell..

[65] Wallot-Hieke N., Verma N., Schlutermann D., Berleth N., Deitersen J., Bohler P. (2018). Systematic analysis of ATG13 domain requirements for autophagy induction. Autophagy.

[66] Gu Y., Desai A., Corbett K.D. (2022). Evolutionary dynamics and molecular mechanisms of HORMA domain protein signaling. Annu. Rev. Biochem..

[67] Nguyen A., Lugarini F., David C., Hosnani P., Alagoz C., Friedrich A. (2023). Metamorphic proteins at the basis of human autophagy initiation and lipid transfer. Mol. Cell..

[68] Ren X., Nguyen T.N., Lam W.K., Buffalo C.Z., Lazarou M., Yokom A.L. (2023). Structural basis for ATG9A recruitment to the ULK1 complex in mitophagy initiation. Sci. Adv..

[69] Bach M., Larance M., James D.E., Ramm G. (2011). The serine/threonine kinase ULK1 is a target of multiple phosphorylation events. Biochem. J..

[70] Egan D.F., Chun M.G., Vamos M., Zou H., Rong J., Miller C.J. (2015). Small molecule inhibition of the autophagy kinase ULK1 and identification of ULK1 substrates. Mol. Cell..

[71] Joo J.H., Dorsey F.C., Joshi A., Hennessy-Walters K.M., Rose K.L., McCastlain K. (2011). Hsp90-Cdc37 chaperone complex regulates Ulk1- and Atg13-mediated mitophagy. Mol. Cell,.

[72] Pareek G., Kundu M. (2024). Physiological functions of ULK1/2. J. Mol. Biol..

[73] Russell R.C., Tian Y., Yuan H., Park H.W., Chang Y.Y., Kim J. (2013). ULK1 induces autophagy by phosphorylating Beclin-1 and activating VPS34 lipid kinase. Nat. Cell, Biol..

[74] Wold M.S., Lim J., Lachance V., Deng Z., Yue Z. (2016). ULK1-mediated phosphorylation of ATG14 promotes autophagy and is impaired in Huntington's disease models. Mol. Neurodegener..

[75] Di Bartolomeo S., Corazzari M., Nazio F., Oliverio S., Lisi G., Antonioli M. (2010). The dynamic interaction of AMBRA1 with the dynein motor complex regulates mammalian autophagy. J. Cell, Biol..

[76] Zhou C., Ma K., Gao R., Mu C., Chen L., Liu Q. (2017). Regulation of mATG9 trafficking by Src- and ULK1-mediated phosphorylation in basal and starvation-induced autophagy. Cell, Res,.

[77] Pengo N., Agrotis A., Prak K., Jones J., Ketteler R. (2017). A reversible phospho-switch mediated by ULK1 regulates the activity of autophagy protease ATG4B. Nat. Commun..

[78] Egan D.F., Shackelford D.B., Mihaylova M.M., Gelino S., Kohnz R.A., Mair W. (2011). Phosphorylation of ULK1 (hATG1) by AMP-activated protein kinase connects energy sensing to mitophagy. Science.

[79] Longo M., Bishnu A., Risiglione P., Montava-Garriga L., Cuenco J., Sakamoto K. (2024). Opposing roles for AMPK in regulating distinct mitophagy pathways. Mol. Cell..

[80] Lin S.Y., Li T.Y., Liu Q., Zhang C., Li X., Chen Y. (2012). GSK3-TIP60-ULK1 signaling pathway links growth factor deprivation to autophagy. Science.

[81] Puente C., Hendrickson R.C., Jiang X. (2016). Nutrient-regulated phosphorylation of ATG13 inhibits starvation-induced autophagy. J. Biol. Chem..

[82] Dunlop E.A., Hunt D.K., Acosta-Jaquez H.A., Fingar D.C., Tee A.R. (2011). ULK1 inhibits mTORC1 signaling, promotes multisite Raptor phosphorylation and hinders substrate binding. Autophagy.

[83] Jung C.H., Seo M., Otto N.M., Kim D.H. (2011). ULK1 inhibits the kinase activity of mTORC1 and cell proliferation. Autophagy.

[84] Du Z., Lovly C.M. (2018). Mechanisms of receptor tyrosine kinase activation in cancer. Mol. Cancer..

[85] Yeh Y.Y., Shah K.H., Herman P.K. (2011). An Atg13 protein-mediated self-association of the Atg1 protein kinase is important for the induction of autophagy. J. Biol. Chem..

[86] Cook A.S.I., Chen M., Nguyen T.N., Cabezudo A.C., Khuu G., Rao S. (2025). Structural pathway for PI3-kinase regulation by VPS15 in autophagy. Science.

[87] Tabata K., Imai K., Fukuda K., Yamamoto K., Kunugi H., Fujita T. (2024). Palmitoylation of ULK1 by ZDHHC13 plays a crucial role in autophagy. Nat. Commun..

[88] Ohashi Y., Tremel S., Williams R.L. (2019). VPS34 complexes from a structural perspective. J. Lipid. Res..

[89] Auger K.R., Carpenter C.L., Cantley L.C., Varticovski L. (1989). Phosphatidylinositol 3-kinase and its novel product, phosphatidylinositol 3-phosphate, are present in Saccharomyces cerevisiae. J. Biol. Chem..

[90] Herman P.K., Emr S.D. (1990). Characterization of VPS34, a gene required for vacuolar protein sorting and vacuole segregation in Saccharomyces cerevisiae. Mol. Cell. Biol..

[91] Schu P.V., Takegawa K., Fry M.J., Stack J.H., Waterfield M.D., Emr S.D. (1993). Phosphatidylinositol 3-kinase encoded by yeast VPS34 gene essential for protein sorting. Science.

[92] Bilanges B., Posor Y., Vanhaesebroeck B. (2019). PI3K isoforms in cell signalling and vesicle trafficking. Nat. Rev. Mol. Cell. Biol..

[93] Backer J.M. (2016). The intricate regulation and complex functions of the Class III phosphoinositide 3-kinase Vps34. Biochem. J..

[94] Herman P.K., Stack J.H., Emr S.D. (1991). A genetic and structural analysis of the yeast Vps15 protein kinase: evidence for a direct role of Vps15p in vacuolar protein delivery. EMBO J..

[95] Stack J.H., Herman P.K., Schu P.V., Emr S.D. (1993). A membrane-associated complex containing the Vps15 protein kinase and the Vps34 PI 3-kinase is essential for protein sorting to the yeast lysosome-like vacuole. EMBO J..

[96] Volinia S., Dhand R., Vanhaesebroeck B., MacDougall L.K., Stein R., Zvelebil M.J. (1995). A human phosphatidylinositol 3-kinase complex related to the yeast Vps34p-Vps15p protein sorting system. EMBO J..

[97] Stjepanovic G., Baskaran S., Lin M.G., Hurley J.H. (2017). Vps34 kinase domain dynamics regulate the autophagic PI 3-kinase complex. Mol. Cell..

[98] Liang X.H., Jackson S., Seaman M., Brown K., Kempkes B., Hibshoosh H. (1999). Induction of autophagy and inhibition of tumorigenesis by beclin 1. Nature.

[99] Itakura E., Kishi C., Inoue K., Mizushima N. (2008). Beclin 1 forms two distinct phosphatidylinositol 3-kinase complexes with mammalian Atg14 and UVRAG. Mol. Biol. Cell..

[100] Chang C., Young L.N., Morris K.L., von Bulow S., Schoneberg J., Yamamoto-Imoto H. (2019). Bidirectional control of autophagy by BECN1 BARA domain dynamics. Mol. Cell..

[101] Cianfanelli V., D'Orazio M., Cecconi F. (2015). AMBRA1 and BECLIN 1 interplay in the crosstalk between autophagy and cell proliferation. Cell. Cycle..

[102] Liang X.H., Kleeman L.K., Jiang H.H., Gordon G., Goldman J.E., Berry G. (1998). Protection against fatal Sindbis virus encephalitis by beclin, a novel Bcl-2-interacting protein. J. Virol..

[103] Kihara A., Noda T., Ishihara N., Ohsumi Y. (2001). Two distinct Vps34 phosphatidylinositol 3-kinase complexes function in autophagy and carboxypeptidase Y sorting in Saccharomyces cerevisiae. J. Cell. Biol..

[104] Matsunaga K., Saitoh T., Tabata K., Omori H., Satoh T., Kurotori N. (2009). Two Beclin 1-binding proteins, Atg14L and Rubicon, reciprocally regulate autophagy at different stages. Nat. Cell. Biol..

[105] Miller S., Tavshanjian B., Oleksy A., Perisic O., Houseman B.T., Shokat K.M. (2010). Shaping development of autophagy inhibitors with the structure of the lipid kinase Vps34. Science.

[106] Rostislavleva K., Soler N., Ohashi Y., Zhang L., Pardon E., Burke J.E. (2015). Structure and flexibility of the endosomal Vps34 complex reveals the basis of its function on membranes. Science.

[107] Baskaran S., Carlson L.A., Stjepanovic G., Young L.N., Kim D.J., Grob P. (2014). Architecture and dynamics of the autophagic phosphatidylinositol 3-kinase complex. Elife.

[108] Ma M., Liu J.J., Li Y., Huang Y., Ta N., Chen Y. (2017). Cryo-EM structure and biochemical analysis reveal the basis of the functional difference between human PI3KC3-C1 and -C2. Cell. Res.

[109] Young L.N., Goerdeler F., Hurley J.H. (2019). Structural pathway for allosteric activation of the autophagic PI 3-kinase complex I. Proc. Natl. Acad. Sci. U. S. A..

[110] Panaretou C., Domin J., Cockcroft S., Waterfield M.D. (1997). Characterization of p150, an adaptor protein for the human phosphatidylinositol (PtdIns) 3-kinase. Substrate presentation by phosphatidylinositol transfer protein to the p150.Ptdins 3-kinase complex. J. Biol. Chem..

[111] Tanaka T., Ames J.B., Harvey T.S., Stryer L., Ikura M. (1995). Sequestration of the membrane-targeting myristoyl group of recoverin in the calcium-free state. Nature.

[112] Ames J.B., Porumb T., Tanaka T., Ikura M., Stryer L. (1995). Amino-terminal myristoylation induces cooperative calcium binding to recoverin. J. Biol. Chem..

[113] Goldberg J. (1998). Structural basis for activation of ARF GTPase: mechanisms of guanine nucleotide exchange and GTP-myristoyl switching. Cell.

[114] Liu Y., Kahn R.A., Prestegard J.H. (2009). Structure and membrane interaction of myristoylated ARF1. Structure.

[115] Huang W., Choi W., Hu W., Mi N., Guo Q., Ma M. (2012). Crystal structure and biochemical analyses reveal Beclin 1 as a novel membrane binding protein. Cell. Res..

[116] Klionsky D.J., Hurley J.H. (2012). Self-eating with your fingers. Cell. Res..

[117] Fan W., Nassiri A., Zhong Q. (2011). Autophagosome targeting and membrane curvature sensing by Barkor/Atg14(L). Proc. Natl. Acad. Sci. U. S. A..

[118] Vanni S., Vamparys L., Gautier R., Drin G., Etchebest C., Fuchs P.F. (2013). Amphipathic lipid packing sensor motifs: probing bilayer defects with hydrophobic residues. Biophys. J..

[119] Ohashi Y., Tremel S., Masson G.R., McGinney L., Boulanger J., Rostislavleva K. (2020). Membrane characteristics tune activities of endosomal and autophagic human VPS34 complexes. Elife.

[120] Tremel S., Ohashi Y., Morado D.R., Bertram J., Perisic O., Brandt L.T.L. (2021). Structural basis for VPS34 kinase activation by Rab1 and Rab5 on membranes. Nat. Commun..

[121] Araki Y., Ku W.C., Akioka M., May A.I., Hayashi Y., Arisaka F. (2013). Atg38 is required for autophagy-specific phosphatidylinositol 3-kinase complex integrity. J. Cell. Biol..

[122] Young L.N., Cho K., Lawrence R., Zoncu R., Hurley J.H. (2016). Dynamics and architecture of the NRBF2-containing phosphatidylinositol 3-kinase complex I of autophagy. Proc. Natl. Acad. Sci. U. S. A..

[123] Cao Y., Wang Y., Abi Saab W.F., Yang F., Pessin J.E., Backer J.M. (2014). NRBF2 regulates macroautophagy as a component of Vps34 Complex I. Biochem. J..

[124] Lu J., He L., Behrends C., Araki M., Araki K., Jun Wang Q. (2014). NRBF2 regulates autophagy and prevents liver injury by modulating Atg14L-linked phosphatidylinositol-3 kinase III activity. Nat. Commun..

[125] Ohashi Y., Soler N., Garcia Ortegon M., Zhang L., Kirsten M.L., Perisic O. (2016). Characterization of Atg38 and NRBF2, a fifth subunit of the autophagic Vps34/PIK3C3 complex. Autophagy.

[126] Zhong Y., Morris D.H., Jin L., Patel M.S., Karunakaran S.K., Fu Y.J. (2014). Nrbf2 protein suppresses autophagy by modulating Atg14L protein-containing Beclin 1-Vps34 complex architecture and reducing intracellular phosphatidylinositol-3 phosphate levels. J. Biol. Chem..

[127] Lee E.F., Perugini M.A., Pettikiriarachchi A., Evangelista M., Keizer D.W., Yao S. (2016). The BECN1 N-terminal domain is intrinsically disordered. Autophagy.

[128] Oberstein A., Jeffrey P.D., Shi Y. (2007). Crystal structure of the Bcl-XL-Beclin 1 peptide complex: beclin 1 is a novel BH3-only protein. J. Biol. Chem..

[129] Pattingre S., Tassa A., Qu X., Garuti R., Liang X.H., Mizushima N. (2005). Bcl-2 antiapoptotic proteins inhibit Beclin 1-dependent autophagy. Cell.

[130] Sinha S., Levine B. (2008). The autophagy effector Beclin 1: a novel BH3-only protein. Oncogene.

[131] Wei Y., Pattingre S., Sinha S., Bassik M., Levine B. (2008). JNK1-mediated phosphorylation of Bcl-2 regulates starvation-induced autophagy. Mol. Cell..

[132] Marquez R.T., Xu L. (2012). Bcl-2:Beclin 1 complex: multiple, mechanisms regulating autophagy/apoptosis toggle switch. Am. J. Cancer. Res..

[133] Mei Y., Glover K., Su M., Sinha S.C. (2016). Conformational flexibility of BECN1: essential to its key role in autophagy and beyond. Protein. Sci..

[134] Mukhopadhyay S., Subedi S., Hopkins J.B., Ugrinov A., Chakravarthy S., Colbert C.L. (2024). Invariant BECN1 CXXC motifs bind Zn(2+) and regulate structure and function of the BECN1 intrinsically disordered region. Autophagy.

[135] Matsunaga K., Morita E., Saitoh T., Akira S., Ktistakis N.T., Izumi T. (2010). Autophagy requires endoplasmic reticulum targeting of the PI3-kinase complex via Atg14L. J. Cell. Biol..

[136] Sun T., Li X., Zhang P., Chen W.D., Zhang H.L., Li D.D. (2015). Acetylation of Beclin 1 inhibits autophagosome maturation and promotes tumour growth. Nat. Commun..

[137] Su H., Yang F., Wang Q., Shen Q., Huang J., Peng C. (2017). VPS34 acetylation controls its lipid kinase activity and the initiation of canonical and non-canonical autophagy. Mol. Cell..

[138] Guo R., Liu J., Min X., Zeng W., Shan B., Zhang M. (2024). Reduction of DHHC5-mediated beclin 1 S-palmitoylation underlies autophagy decline in aging. Nat. Struct. Mol. Biol..

[139] Yang Y., Fiskus W., Yong B., Atadja P., Takahashi Y., Pandita T.K. (2013). Acetylated hsp70 and KAP1-mediated Vps34 SUMOylation is required for autophagosome creation in autophagy. Proc. Natl. Acad. Sci. U. S. A..

[140] Dossou A.S., Basu A. (2019). The emerging roles of mTORC1 in macromanaging autophagy. Cancers (Basel).

[141] Mercer T.J., Gubas A., Tooze S.A. (2018). A molecular perspective of mammalian autophagosome biogenesis. J. Biol. Chem..

[142] Kim J., Kim Y.C., Fang C., Russell R.C., Kim J.H., Fan W. (2013). Differential regulation of distinct Vps34 complexes by AMPK in nutrient stress and autophagy. Cell.

[143] Wang R.C., Wei Y., An Z., Zou Z., Xiao G., Bhagat G. (2012). Akt-mediated regulation of autophagy and tumorigenesis through Beclin 1 phosphorylation. Science.

[144] Zalckvar E., Berissi H., Mizrachy L., Idelchuk Y., Koren I., Eisenstein M. (2009). DAP-kinase-mediated phosphorylation on the BH3 domain of beclin 1 promotes dissociation of beclin 1 from Bcl-XL and induction of autophagy. EMBO Rep..

[145] Erlich S., Mizrachy L., Segev O., Lindenboim L., Zmira O., Adi-Harel S. (2007). Differential interactions between Beclin 1 and Bcl-2 family members. Autophagy.

[146] Strappazzon F., Vietri-Rudan M., Campello S., Nazio F., Florenzano F., Fimia G.M. (2011). Mitochondrial BCL-2 inhibits AMBRA1-induced autophagy. EMBO J..

[147] Fimia G.M., Stoykova A., Romagnoli A., Giunta L., Di Bartolomeo S., Nardacci R. (2007). Ambra1 regulates autophagy and development of the nervous system. Nature.

[148] Molejon M.I., Ropolo A., Re A.L., Boggio V., Vaccaro M.I. (2013). The VMP1-Beclin 1 interaction regulates autophagy induction. Sci. Rep..

[149] Tabara L.C., Escalante R. (2016). VMP1 establishes ER-microdomains that regulate membrane contact sites and autophagy. PLoS One.

[150] Park J.M., Jung C.H., Seo M., Otto N.M., Grunwald D., Kim K.H. (2016). The ULK1 complex mediates MTORC1 signaling to the autophagy initiation machinery via binding and phosphorylating ATG14. Autophagy.

[151] Park J.M., Seo M., Jung C.H., Grunwald D., Stone M., Otto N.M. (2018). ULK1 phosphorylates Ser30 of BECN1 in association with ATG14 to stimulate autophagy induction. Autophagy.

[152] Mercer T.J., Ohashi Y., Boeing S., Jefferies H.B.J., De Tito S., Flynn H. (2021). Phosphoproteomic identification of ULK substrates reveals VPS15-dependent ULK/VPS34 interplay in the regulation of autophagy. EMBO J..

[153] Banerjee C., Mehra D., Song D., Mancebo A., Park J.M., Kim D.H. (2023). ULK1 forms distinct oligomeric states and nanoscopic structures during autophagy initiation. Sci. Adv..

[154] Joachim J., Jefferies H.B., Razi M., Frith D., Snijders A.P., Chakravarty P. (2015). Activation of ULK kinase and autophagy by GABARAP trafficking from the centrosome is regulated by WAC and GM130. Mol. Cell..

[155] Fracchiolla D., Chang C., Hurley J.H., Martens S. (2020). A PI3K-WIPI2 positive feedback loop allosterically activates LC3 lipidation in autophagy. J. Cell. Biol..

